# Blocking *Plasmodium falciparum* Malaria Transmission with Drugs: The Gametocytocidal and Sporontocidal Properties of Current and Prospective Antimalarials

**DOI:** 10.3390/ph4010044

**Published:** 2010-12-23

**Authors:** Anthony E. Kiszewski

**Affiliations:** Department of Natural and Applied Sciences, Bentley University, Waltham, MA 02452, USA; E-Mail: akiszewski@bentley.edu; Tel.: +1-781-891-2954; Fax: +1-781-891-2838

**Keywords:** *Plasmodium*, gametocyte, sporogony, antimalarials, transmission-blocking

## Abstract

Drugs that kill or inhibit the sexual stages of *Plasmodium* could potentially amplify or synergize the impact of other interventions by blocking transmission to mosquitoes. Primaquine and other 8-aminoquinolines have long offered such potential, but safety and other concerns have limited their use. Although transmission-blocking properties are not often a priority of drug discovery efforts, a number of interesting gametocytocidal and/or sporontocidal drug candidates have emerged in recent years. Some still bear significant technical and safety concerns, while others have passed clinical trials and are on the verge of entering the antimalarial armamentarium. Recent advances in our knowledge of gametocyte differentiation, gametogenesis and sporogony have also led to the identification of a large array of potential new targets for drugs that might interfere with malaria transmission. This review examines the properties of existing and prospective drugs, mechanisms of action, counter-indications and their potential role in regional malaria elimination efforts.

## 1. Introduction

Gametocytes, the sole stage of malaria parasites capable of infecting mosquitoes, represent a key link in human/vector malaria transmission. Preventing or reducing the development, function, survival or carriage of gametocytes in human hosts can interrupt transmission. As we await the development of an effective transmission-blocking vaccine [1], some existing drugs and several in the advanced stages of development already demonstrate such capabilities.

Most drug discovery efforts have focused on the asexual erythrocytic stages that cause the acute symptoms of malaria. Relatively few extant or prospective antimalarial therapies significantly impair the development or survival of *Plasmodium falciparum* gametocytes. Chloroquine, for example, eliminated asexual parasites and immature gametocytes, but left treated individuals fully capable of transmitting infections [2], even before resistance to it became widespread.

As would be the case with a transmission-blocking vaccine [1], a drug that theoretically only killed gametocytes or inhibited sporogony [3] would confer no direct benefit to a person infected with malaria. They would simply reduce the chances that an infected person might pass on infections to mosquitoes and thus, to other members of the community. This could in turn benefit individuals by reducing the chances of re-infection and decreasing the frequency of severe or complicated malaria by limiting the clonal complexity of superinfections [4].

From a community perspective, drugs that block transmission could prove powerfully complementary when combined with other interventions. Reduced transmission would ease case loads at treatment facilities, increasing availability of resources for those who do become infected. Transmission-blocking drugs could thus provide welcome synergies in an integrated program of antimalarial intervention, particularly in an era with ambitions for local and regional elimination of transmission.

Transmission blocking properties against *P. falciparum* are well known in the existing antimalarial armamentarium and have been observed and reported in many prospective drugs, particularly against animal models *in vitro* [3]. Relatively few prospects, however, have gone on to human clinical trials and further development into commercial therapies. Many prove overly toxic, insufficiently bioavailable or metabolically stable, or have simply been neglected.

Nevertheless, a number of promising drugs, both novel and derived from existing classes of antimalarial drugs, are currently under active development. This review examines the nature of some of the more promising transmission-blocking drugs in various stages of the developmental pipeline, their modes of action against gametocytes, their prospects and limitations.

## 2. Gametocytogenesis

Gametocytes are the haploid sexual stages of *Plasmodium* parasites, precursors to the gametes that initiate sporogony in the midguts of mosquito vectors. Gametocytogenesis, the process by which they are formed, provides targets for drugs that might interfere with their production. In *P. falciparum* gametocytes derive directly from the asexual erythrocytic merozoites present in the peripheral blood of a person infected with malaria. Sexual determination is apparently pre-determined in the asexual stage as a single schizont gives rise to only male or female gametocytes [5,6].

Gametocytes appear generally 10-14 days after the appearance of asexual parasites in erythrocytes [7] through the process of gametocytogenesis. Only a small percentage of merozoites develop into gametocytes, in some estimates, less than 1% [8].

These haploid male and female gametocytes, which are the precursors of the gametes that will undergo syngamy (fertilization) in the invertebrate host, but only after they complete a maturation process or capacitation that can require up to 8-10 days to complete in *P. falciparum* [8,9]. Gametocytes can be carried by infected individuals for months (mean = 55 days) although the average circulation time per individual gametocyte has been estimated between 4.6-6.5 days [10]. Infections that are clonally diverse have been associated with longer durations of gametocytemia [11].

The maturation process can be divided into five morphological stages [12,13] (Table 1). Prior to maturation, gametocytes are non-infectious. Stages I-IV are sequestered in capillaries while they develop, entering the peripheral circulation only upon maturation to Stage V.

## 3. General Strategies of Transmission-Blocking

Although new targets and strategies of attack continue to be identified, the basic and common modes of sexual stage interference for existing drugs include:

### 3.1. Inhibition of Metabolic Processes in Early Gametocytogenesis

Developing gametocytes (Stages I-III) are particularly vulnerable to drugs that affect hemoglobin metabolism [3]. Artemisinins, for example, inhibit both heme polymerization and the hemoglobin catabolic pathway [16]. Certain new targets attack other pathways essential to the transition from asexual to sexual stages.

### 3.2. Toxicity against Mature Gametocytes

Later stage gametocytes (Stages IV-V), which are more quiescent metabolically, are more likely to be affected by drugs with cytotoxic properties. Some 8-aminoquinolines (8AQ) including primaquine may directly exert toxicity against parasite mitochondria, inhibiting metabolism through interference with the electron transport functions of ubiquinone in the respiratory chain [17]. Certain 8AQ metabolites may also kill by inducing oxidative stress within cells through the spontaneous generation of superoxides [18,19]. Toxicity of several drugs, including atovaquone, may relate to their activity against the dihydroorotate dehydrogenase complex of mitochondria, which functions in the biosynthesis of pyrimidines [3]. The array of targets available for inducing parasite-specific toxicity continues to expand and will be discussed in detail in later sections.

### 3.3. Inhibition or Disruption of Gametogenesis and/or Sporogony

Diverse mechanisms are involved in interrupting transmission after gametocytogenesis, some operating along the various stages of extrinsic incubation from exflagellation to the formation of sporozoites, or even by damaging the salivary glands of vector mosquitoes [3]. Pyrimethamine has been shown to directly damage ookinetes and along with proguanil, inhibit dihydrofolate reductase [3]. New opportunities for interference with gametogenesis and sporogony have arisen, particularly with regard to proteases and protein kinases. Many of the molecular mechanisms involved in the transition through sporogony remain to be determined.

Drugs that are also highly effective against asexual parasites would be most attractive for incorporation into front-line therapies. Alternatively, a transmission blocker can be incorporated as part of a combination or used as a follow-on drug as part of a course of treatment that would both treat clinical infections and prevent the transmission of parasites to others.

## 4. Primaquine and Other Established 8-Aminoquinolines

Primaquine, an 8-aminoquinoline (along with pamaquine), has long been known to reduce the prevalence of gametocytes circulating in the peripheral bloodstream of parasitemic individuals and to prevent exflagellation in gametocytes that are present [20]. Primaquine has no sporontocidal effect on *P. falciparum,* however [21]. The related 4-aminoquinolines, including chloroquine, affect immature gametocytes of *P. falciparum*, but not mature gametocytes [22]. For decades, primaquine had been the only significant gametocytocidal drug in the malaria armamentarium.

In the body, primaquine is quickly metabolized via oxidative deamination at the C-4′ into its primary metabolite carboxyprimaquine which displays much reduced antimalarial activity [23,24]. Other metabolites vary in their antimalarial activity, with 5,6-dihydroxy-8-aminoquinoline showing relatively intense *in vitro* inhibition of *P. berghei* exoerythrocytic stages while 6-hydroxy-8-amino-quinoline is even less active than carboxyprimaquine [24]. Metabolites that did not spontaneously generate superoxides were the least active against parasites. Primaquine's relatively short half-life of about 7 h [25] needs to be considered in dosing to optimize and/or sustain its effects over a period sufficient to clear the bloodstream of gametocytes.

Studies suggest that a single adult dose of 30-45 mg primaquine (0.50-0.75 mg base/kg in children) for interrupting transmission may be more beneficial if given two weeks post ACT treatment immediately after treatment because additional gametocytes may continue to develop from residual asexual parasites until 7-15 days after completion of a course of ACT [25].

The efficacy of primaquine has inspired researchers to seek analogs that may exhibit similar potency against gametocytes, including certain dipeptide derivatives that show similar levels of activity [23]. Its gametocytocidal activity appears to be associated with the presence of a terminal amino group in these primaquine analogs. Acylation of the aliphatic side-chains of primaquine derivatives effectively blocks their conversion to carboxyprimaquine, extending their activity.

Despite their early promise, these primaquine derivatives have never been fully developed as therapies. Adverse interactions between primaquine and G6PD-deficient (G6PDd) individuals, are a major limitation of this approach, and may have discouraged the further development of such therapeutics.

Toxic metabolites of primaquine including 5-hydroxyprimaquine and 6-methoxy-8-aminoquinoline [26] may induce a severe hemolytic anemia by oxidizing glutathione to glutathione disulfide which leaches from red cells and lead to denatured, insoluble aggregates of hemoglobin in intact cells that are then preferentially removed from circulation by the spleen and liver [27].

Although these risks are sometimes downplayed based on the relatively benign findings of clinical studies performed in the 1950s, prudence demands caution because these studies involved a variant of G6PDd (African A-) that involves a relatively mild manifestation of primaquine sensitivity [28]. Thus, primaquine may be contraindicated in areas (such as most of Africa) where G6PDd is prevalent, including most of Africa, where screening would be necessary before administering primaquine. G6PD-deficiency is not a significant issue in South America or Asia, however.

## 5. Gametocytocidal Effects of Artemisinins and ACTs

Gametocytes are refractory to many of the early front-line therapies, including chloroquine, thus may continue to circulate for weeks to months after all asexual parasites have been eliminated from the bloodstream after treatment [10]. Most ACTs, however, the current choice for treating malaria contain an artemisinin derivative that is active against gametocytes.

Artemisinins act primarily on younger gametocytes [29,30], inhibiting differentiation to the mature infective stages. ACTs also shorten the typical carriage of gametocytes in the blood from 55.6 days in patients receiving non-ACT therapies to 13.4-28.6 days in those receiving ACTs [10].

Certain studies, however, have demonstrated activity against mature gametocytes *in vitro* [31,32], though this effect does not appear to translate to significant clinical impacts against mature gametocytes in infected patients.

A typical course of ACT also does not result in complete clearance of developing gametocytes [33]. Gametocytes were 3-fold less prevalent and 5-6 fold less abundant in individuals, although children remained infectious to mosquitoes [33]. The probability of transmission in children treated with artesunate and SP was one ninth that in children treated with SP alone (0.3% to 2.7%) [33]. Thus, transmission is not completely blocked by treatment with ACTs, which can pose a problem in areas with high vectorial capacity.

The gametocytocidal properties can be effectively complemented by adding a single dose of primaquine to the end of a course of ACT [25]. This approach could also be employed with one of newer gametocytocidal drugs, as they become available.

Pyronaridine, an established antimalarial drug that had been in clinical use in China since the 1980s [34], may offer another currently available option. This compound is derived from mepacrine, one of the first synthetic antimalarials, and bears a side-chain similar to that in amodiaquine. It shows high efficacy and tolerance in clinical trials, but also a high likelihood of resistance if employed as a monotherapy.

Information on the gametocytocidal properties of pyronaridine, however, is limited and conflicting. Although an *in vitro* study against isolates of multidrug-resistant *P. falciparum* showed potent schizontocidal and gametocytocidal properties (LC50, 6-20 nM) [35], no such activity was noted in a study of patients with uncomplicated *falciparum* malaria [36]. In any case, pyronaridine, as part of a combination therapy that includes artesunate (Pyramax), has successfully completed Phase III clinical trials against *P. falciparum* [37].

## 6. Gametocytocidal Therapies under Development

A relatively small proportion of the antimalarials currently under development [38,39] exhibit gametocytocidal or sporontocidal properties. This section examines both some of the more promising candidates under active development as well as several moieties that remain promising but whose development for reasons not necessarily related to their potential utility. Although the focus is on drugs active against *P. falciparum*, drugs shown to be efficacious against *P. vivax* gametocytes or those of murine and simian models of malaria are also considered, as they may have potential against *P. falciparum*.

### 6.1. Newer 8-Aminoquinolines

#### Tafenoquine (WR-238605)

Tafenoquine is an 8-aminoquinoline relative of primaquine that has a much longer metabolic half-life (two-weeks). It is currently in the final stages of development and is undergoing Phase IIb/III clinical trials.

It is unique among antimalarials in exhibiting activity against all stages of the parasite, including liver schizonts. It appears to be less toxic and more clinically effective than primaquine [40], although it is counter-indicated in pregnant women and individuals with G6PDd [41].

The extended presence and activity of tafenoquine has profound implications regarding its utility as a transmission-blocking drug. It is retained in the body at functional doses for the entire period required for asexual parasites to develop into mature gametocytes, thus facilitating clearance of all infective parasites.

Against *P. vivax,* tafenoquine was found to inhibit sporogonic development in *An. dirus* at a dose of 25 mg/kg [42,43]. It appears to be effective at inhibiting sporogony in mosquitoes even when mosquitoes are exposed to it four days after acquiring an infection. Its presence did not eliminate all oocysts or sporozoites, though its effect on delaying development can theoretically still impose a strong effect on transmission by allowing attrition to take an extra toll on infected mosquitoes during extrinsic incubation, thus decreasing vectorial capacity. 6.1.2. Elubaquine (Bulaquine)

Elubaquine is a synthetic 8-aminoquinoline is differentiated from primaquine by a 2,4 dihydrofuran side chain in the 8 position of the quinoline. It was found to be gametocytocidal against *P. cynomolgi* in rhesus monkeys [44] but did not inhibit sporogony. It has also undergone clinical trials in human subjects against *P. vivax* in Thailand [45] as an anti-relapse therapy and found to be comparable to primaquine in terms of safety and tolerability. Elubaquine was found to be superior to primaquine in clearing gametocytemias of *P. falciparum* in patients with acute infections over an extended period (8 days) [46].

#### 6.2. Trioxaquines

Trioxaquines are essentially hybrid molecules containing synthetic peroxides. They were developed through a rational approach intended to mimic the effect of the 1,2,4-trioxane groups of artemisinins that damage parasites via the production of free radicals and to enhance its pharmacokinetics [47]. Trioxaquines are, thus, covalent chimers combining a parasite-toxic trioxane to an aminoquinoline that bioaccumulates within parasites.

Trioxolaquines, related compounds incorporating trioxolane moieties, though active against malaria parasites, appear to be less promising as developmental candidates due to their lower metabolic stability [48].

One of the more active versions of the trioxaquines appears to be trioxaquine 2 (DU1302), a salt formed by the protonation of trioxaquine citric acid [49]. *In vitro* tests against *P. falciparum* revealed this compound to be highly active both against young gametocytes (Stages II-IV) as well as mature, Stage V gametocytes, showing a level of activity (IC_50_: 46-108 nM) somewhat greater than that exhibited by artesunate.

This anti-gametocyte activity, however, was up to 1,100 fold greater than that demonstrated by the nonperoxidic antimalarials chroloquine, primaquine and atovaquone. The effectiveness of DU1302 was not affected by parasite chloroquine resistance, and was independent of stereochemistry, with both isomers exhibiting equal activity. Yet another trioxaquine moiety, PA1103/SAR116242, appears even more active against *P. falciparum*, demonstrating an IC_50_ value of 10 nm against FcM29, a choroquine-resistant strain [48]. Absorption, safety and metabolic parameters appear particularly favorable for further development of this drug.

Trioxaquines have not yet been evaluated against *P. falciparum in vivo* in humans, but show promise against *P. falciparum* infecting humanized mice [48]. An oral dose of 63 mg/kg per day of PA1103/SAR116242 induced a complete cure in all treated mice. Toxicity levels are relatively low compared to some prospective therapies, with an estimated therapeutic index (TI) of 23 for trioxaquine-2 and 100 for trioxaquine-4 [49].

### 6.3. Epoxomicin

Epoxomicin is naturally derived from certain strains of *Actinomycetes* bacteria, originally evaluated for its anti-tumor properties [50]. Its active moiety appears to be an α9,β9-epoxyketone. It can also be synthesized from spirodiepoxides [51]. It acts as a proteasome inhibitor, exploiting the expression of genes that encode cysteine proteases and the proteasome throughout gametocytogenesis [52]. The proteasome is a complex of nuclear proteases responsible for degrading and recycling unnecessary or damaged intracellular proteins.

A broad *in vitro* screening of potential proteasome inhibitors revealed that 100 nM of epoxomicin, was found to reduce *P. falciparum* gametocytes by 77% within 24 h. Complete kill of gametocytes was achieved after 72 h, even at a dose of 10 nM [52].

This inhibition of proteasome activity appears to have a toxic affect, and does not lead to resumption of development if inhibition is removed. Specifically, epoxomicin interacts with the β5 and β2 subunits of the *P. falciparum* proteasome. While these active sites are conserved, the amino acids surrounding them are distinct in *P. falciparum,* suggesting targets for novel inhibitors that preferentially block *P. falciparum* enzymes. Epoxomicin did not appear to adversely affect the viability of mouse (3T3) or human (A549) cell lines, offering promise for future *in vivo* investigations.

Epoxomicin also affects the morphology of developing gametocytes, greatly restricting the circumference of parasites exposed to this compound. Mature stage V gametocytes, however, are not inhibited from undergoing exflagellation *in vitro*.

Another proteasome inhibitor evaluated, *N*-acetyl-l-leucyl-l-leucyl-l-methioninal (ALLN), was also effective at killing gametocytes, but only at higher concentrations (100nM) [52].

### 6.4. Methylene Blue (Methylthioninium Chloride)

Methylene Blue (MB) is a heterocyclic aromatic compound with a wide array of uses, including as a bacteriologic stain. Medicinally, it is most commonly used as an antidote to reverse methemoglobinemia in cases of cyanide poisoning. MB was one of the first synthetic drugs ever used against malaria [53]. It acts by inhibiting disulfide reductases and heme detoxification [54].

Methylene Blue given in combination with amodiaquine or artesunate at a daily dose 10 mg/kg for three days, exhibited strong gametocytocidal activity against *P. falciparum in vivo* [55]. Unlike the standard artesunate-amodiaquine (AS-AQ) controls, both MB-AS and MB-AQ achieved complete clearance of all sexual stages. It appears to act against both older gametocytes and those still developing. In terms of PCR-confirmed clinical and parasitological efficacy, MB-AQ outperformed the AS-AQ controls 95% to 82%. As a well-tolerated and highly efficacious gametocytocide, MB shows great potential as a component of prospective combination therapies against malaria.

### 6.5. Newer Artemisinins

#### 6.5.1. Sodium beta-Artelinate

Sodium beta-artelinate is a water soluble, artemisinin analogue synthesized from artemisinin. It has demonstrated potent gametocytocidal but no sporontocidal effects against the simian malaria *P. cynomolgi* [56]. Transmission-blocking properties against *P. falciparum* have not yet been reported.

#### 6.5.2. Alpha/beta arteether

Alpha/beta arteether is an oil soluble ethyl ether derivative of artemisinin that is produced as a racemic mixture of enantiomers. It has passed clinical trials for safety and has been found to be efficacious against the asexual stages of *P. falciparum* [57]. Gametocytocidal activity has been demonstrated against *P. cynomolgi* B [56,58] at a single dose of 10 mg/kg. As with other artemisinin derivatives, there was no sporontocidal effect.

### 6.6. Dyhydroacridine-dione (WR-250547)

Dyhydroacridine-dione administered at a dose of 0.39 mg/kg was found to inhibit sporogonic development in *An. dirus* [42,43]. (WR-250547) It appears to be effective at preventing sporozoites from entering salivary glands even up to eight days after administration. This drug is not currently under development as an antimalarial, despite its promising sporontocidal properties.

### 6.7. Tipranavir (Aptivus)

Tipranavir, a nonpeptidic HIV protease inhibitor was found to be both gametocytocidal and inhibitory of gametocytogenesis at clinically relevant doses (EC_50_, 12 to 21 μM) [59]. Several other protease inhibitors evaluated in the same trial were also found to be inhibitory, including: darunavir, saquinavir and lopinavir.

### 6.8. 9-Anilinoacridines

The 9-anilinoacridines were originally developed as anti-cancer agents [60]. Analogs bearing 3,6-diamino substitutions on their acridine rings are particularly active in inhibiting DNA topoisomerase II and the formation of β-hematin in asexual parasites of *P. falciparum* [61]. However, this 3,6-diamino substitution is less effective against sexual stages. The most effective moiety against asexual stages was 3,000-fold less effective against gametocytes. The most gametocytocidally active of an array of thirteen analogs evaluated *in vitro* against *P. falciparum* was a 1′-CH_2_NMe_2_-9-anilinoacridine (IC_50_, 0.82-2.1 μM) against the KT1 and KT3 strains, respectively [62].

### 6.9. Riboflavin

Riboflavin has been found to be toxic to *P. falciparum* gametocytes *in vitro*, by possibly interfering with the digestion of hemoglobin during gametocytogenesis. It appears to be effective against both immature and mature gametocytes. Riboflavin also potentiates activity against asexual parasites synergistically with drugs including mefloquine, pyrimethamine and quinine [63], suggesting that it may warrant consideration as a component of a combination therapy.

### 6.10. Neem (Azadirachtin and Other Limonoids)

Neem is a product derived from *Azadirachta indica,* a tree native to India. The repellent and insecticidal properties of neem and its derivatives have long been known [64], but also appears to be active against the sexual stages of malaria parasites [65]. The activity of neem, a complex mixture of substances, is attributed to several component limonoids, a class of oxygenated terpenoids, specifically: azadirachtin, gedunin and nibolide [66].

A 50 mg/kg dose of Neemazal®, an azadarachtin-enriched extract of neem seeds, was found to inhibit the development of parasites in *An. stephensi* mosquitoes allowed to feed on rodents infected with *P. berghei* [67]. Neemazal® did not exhibit any observable sporontocidal activity.

Neem leaf extracts have also been shown to be active against the sexual stages of *P. falciparum in vitro* [68], killing more than 90% of both developing and mature gametocytes at a concentration of 2.5 μg/mL [69]. Azadirachtin, in particular, appears to act by preventing exflagellation through inhibition of cytoskeletal activity and interference with microgametocyte endomitosis [70].

While promising, significant technical challenges limit the feasibility of using neem and its components as drugs. Limonoids tend to have minimal bioavailability and suffer from a short metabolic half-life [71]. Gedunin appears to be poorly absorbed in the digestive tract [72]. Once these technical challenges are overcome, questions of safety and interaction with other drugs will remain.

## 7. Potential Targets for Transmission-Blocking Activity

Earlier research on transmission blocking immunity has revealed certain gametocyte-specific proteins associated with surface membranes of gametocytes, including: Pfs230 [73], Pfs48/45 [74] and Pfs25 [75]. Although, the focus of these molecules has been on their potential as antigens for transmission-blocking vaccines, they offer some promise as targets for drug interventions. For example, targeted gene disruption of Pfs230 affects exflagellation and fertilization success [76].

Potential candidates for transmission-blocking drug targets are numerous and growing as our knowledge of the *Plasmodium* proteome and the mechanisms of sexual stage differentiation and transition expands. Molecules suspected or shown to be critical in gametocytogenesis, gametogenesis and sporogony include.

### 7.1. Proteases

Proteolysis is likely to be important both for the remodeling associated with morphological changes during gametocyte formation as well as gamete formation and the invasion of mosquito midgut walls by ookinetes. Thus, proteases may offer a target of drug intervention via inhibitors. In *P. berghei*, for example, both exflagellation center formation can be blocked by 1,10 phenanthroline (1mM) as well as the cysteine/serine protease inhibitors TPCK and TLCK at concentrations of 75 and 100mM [77]. These latter inhibitors also seem to affect ookinete formation while 1,10 phenanthroline has no effect. It may also be worth investigating other protease inhibitors that may have similarly specific effects on certain stages of *P. falciparum*.

Two papain-like cysteine proteases, falcipain-2 [78] and falcipain-3 [79] are present in the food vacuole of trophozoites and young gametocytes and appear to be involved in hemoglobin digestion [80,81]. In late stage gametocytes, only falcipain3 appears to be expressed, and appears to support oocyst development as indicated by the suppressive activity of E64d, a membrane-permeant protease inhibitor [82]. Thus, cysteine proteases appear quite promising as potential targets for the inhibition of gametocytogenesis and sporogony.

### 7.2. Protein Kinases

Protein kinases are involved in the modification of other proteins via phosphorylation. Mitogen-activated protein kinases (MAPKs) are activated by diverse extracellular factors in a protein kinase cascade and appear to be involved in the regulation of the cell cycle [83].

In *P. falciparum*, the novel MAPK Pfmap1 phosphorylates threonine-tyrosine (TXY) sequence motifs as in most eukaryotic MAPKs [84] while Pfmap2 is active against threonine-aspartic acid-tyrosine (TDY) motifs [85]. Pbmap2, the *P. berghei* equivalent of Pfmap2 has been shown to be involved in the regulation of male gametogenesis [86,87], suggesting a similar role for Pfmap2. The divergence of Pfmap2's sequence motif affinity from the typical mammalian pattern offers particular promise as a drug target because of the potential for parasite-selective drug activity which may be associated with low human toxicity.

Similar advantages exist with another group of *P. falciparum*-specific, NIMA-related protein kinases, Pfnek2 [88], Pfnek 3 [89] and Pfnek2 [90]. Disruption of nek-2 and nek-4 genes in *P. berghei* models interferes with DNA replication during meiosis and prevents ookinetes from developing [88,90]. Pfnek3 appears to be a specific activator of the MAPK Pfmap2 [89]. Pfnek3 is expressed in late asexual intraerythrocytic stages as well as in gametocytes.

A calcium-dependent protein kinase (CDPK4) appears to have important role in male gametogenesis and in the maturation of fertilized zygotes [91].

Yet another pair of kinases, MAP2 and NEK4, identified through proteome analysis and investigated through targeted gene disruption [92]. The male gametocytic proteome is particularly rewarding in such searches due to the abundance of distinct proteins involved in flagellar motility and genome replication. MAP2 appears to have a sex-specific role in male gamete formation while NEK4, an FG-specific NIMA-related kinase, appears to be involved in zygote development and meiosis [92].

Thus, protein kinases appear to offer an abundant source of potential targets for transmission-blocking drugs.

### 7.3. Peroxiredoxins

Peroxiredoxins are antioxidant enzymes that protect macromolecules from reactive peroxides and the oxidative stress they impose on sensitive malaria parasites. Certain peroxiredoxins may also be involved in the activation pathways of certain MAP kinases [93].

Gene disruption of the 2-Cys peroxiredoxin TPx-1 in turn disrupts gametocytogenesis in *P. berghei*, making it a useful target for a prospective drug [94]. As has been demonstrated with other molecules, *P. falciparum*-specific analogs of TPx-1 are likely to show similar activity. Sequestration of parasites, more typical of *P. falciparum* infections than in rodent malarias, may exacerbate oxidative stress over what circulating parasites experience, thus increasing their sensitivity to interference with peroxiredoxins [94]. Disruption of TPx-1 did not appear to affect asexual parasite development or male gametogenesis [94].

### 7.4. Effectors of cGMP signaling in gametogenesis

Exflagellation of male gametocytes, triggered in part by the temperature decrease and rise in pH associated with the midgut environment of a mosquito, is mediated by a cascade of factors including a calcium-dependent protein kinase and cGMP [95]. Disruption of a *P. falciparum* gene (PfPDEδ) expressing one of four known cyclic dinucleotide phosphodiesterases (PDE), a key component of the cGMP signaling pathway, interferes with gametogenesis [96]. Similar disruption of guanylyl cyclase, another mediator of cGMP, had no such effect [96]. Three other *P. falciparum* PDE genes remain to be evaluated for similar effects and the role of cGMP in other phases of development may warrant further investigation.

### 7.5. Osmiophilic Bodies and Gamete Emergence

Osmiophilic bodies are granular organelles associated with late stage macrogametocytes of many Apicomplexa [97,98] Their presence appears to be correlated with the emergence of Stage V macrogametocytes from the erythrocytic membrane in the mosquito midgut after the ingestion of infective blood. The main protein associated with osmiophilic bodies, Pfg377, is expressed solely in female gametocytes and appears to play a critical role in this mechanism, as targeted gene disruption reduces the efficiency of female gamete emergence both *in vitro* and *in vivo* [99].

Studies of mutant clones suggest that the *P. falciparum* gene Pf11-1 appears to code for a glutamic acid-rich protein that has a similar and perhaps more general role in the rupture of erythrocytic membranes to allow the escape of gametes [100]. Disruption of these mechanisms would thus inhibit sporogony and transmission.

### 7.6. Other Specific Proteins Upregulated in Gametocytes

Transcriptome analyses [101,102] of parasites undergoing differentiation into gametocytes *in vitro* have been used to reveal a wide array of proteins that are upregulated in the sexual stages. Some of these proteins appear to functionally important in gametocytogenesis, making them potentially useful as targets for drug intervention, although in most cases, their exact role in differentiation and the molecular mechanisms by which they operate require further clarification. Such proteins include:

1) Pfs16 – a membrane protein found in the parasitophorous vacuole of young gametocytes [103,104,105].
2) Pfg27 – a dimeric cytosolic phosphoprotein present during the early formation of gametocytes [106].
3) Pfpeg-3 (Pfmdv-1) – ‘*P. falciparum* protein of early gametocytes.’ encoded by the gene PFL0795c [101,107].
4) Pf peg-4 – a protein encoded by the gene PF10_064 [102].
5) PfPuf1 – an RNA-binding protein upregulated in gametocytes and sporozoites [108].
6) PfPuf2 – an RNA-binding regulatory protein upregulated in gametocytes [109].
7) Pfgig – a gene associated with a protein involved in the transition to early gametocytogenesis via upregulation of Pfs16 [110].
8) HMGB2 – a DNA-binding protein involved in transcriptional regulation of *P. yoelii* [111].
9) Pfg14.744 and Pfg14.748 – an as yet uncharacterized pair of genes expressed in the earliest stages of gametocytogenesis (prior to Stage II) [112].
10) PfRex-3, and PfGEXP10 – proteins of indeterminate function that are both expressed in early gametocytogenesis, cleaved, acetylated and exported into the cytoplasm of infected erythrocytes (along with Pfg14.744) [113].

Pfs16 and Pfg27 are both upregulated during the differentiation of trophozoites to gametocytes prior to the appearance of the subpellicular microtubule network that distinguishes the State II, crescent-shaped gametocytes from earlier forms, suggesting a possible role in inducing these morphological changes [102]. *In vitro* studies suggest that Pfs16 is over-expressed during the first 24 h of culture leading to the production of mature gametocytes [102]. A definitive causal link has not yet been established, however, between this gene or its products and gametocyte maturity [102].

Pfgig is a gene encoding for a yet to be identified protein involved in the transition from asexual stage parasites to sexual stages. Its silencing has a six-fold suppressive effect on gametocyte production and its complementation causes the upregulation of Ps16 [110]. Pfgig is transcribed abundantly in schizonts but no mRNA is detected in gametocytes, suggesting that commitment to sexual stages may occur early in the life cycle of malarial parasites [110].

Both PfPuf1 and PfPuf2 bind RNA and, according to a microarray analysis, are upregulated in gametocytes [109]. A subtraction library analysis suggests that PfPuf1 also appears to be upregulated by sporozoites [114].

PfPuf2 regulates translation in early stage gametocytes, promoting sexual development and sex differentiation [109]. Disrupting the activity of PfPuf2 increases gametocytogenesis and causes distortions in the sex ratio favoring males.

Pfpeg-3, also known as Pfmdv-1 [107] is produced in Stage I gametocytes and present on the gametocyte plasma membrane and other membranes in the parasitophorous vacuole and in the infected erythrocyte. Interference with this protein leads to sex-specific reductions in mature gametocytes, favoring female gametocytes [107]. It has been suggested that disrupted nutrient transport might be responsible for this effect in strains of parasites with a Pfmdv-1 defect [107]. Less is known of Pfpeg-4, other than that it appears in the highest concentrations in Stage II gametocytes [102].

Each of these proteins offer promise as targets for drugs that might disrupt the early stages of gametocytogenesis and skew sex ratios. Expression of HMGB2, a high mobility group protein that binds DNA (but is not sequence-specific) and is conserved in all malaria species, peaks in the gametocyte stage, although translation of mRNA seems to be delayed until parasites have differentiated into ookinetes [111]. Disruption of the locus encoding HMGB2 in *P. yoelii* through gene knockout has been shown to severely impair the formation of oocysts [111]. HMGB2 apparently helps regulate transcription of genes critical for the completion of sporogony.

### 7.7. Receptors and Ligands Involved with Adhesion and Sequestration

Adherence to endothelial tissue beds allows gametocytes to develop in situ without facing the hazards associated with passage through the spleen. This accounts in part for why young gametocytes rarely circulate in peripheral blood and why mature infective gametocytes usually appear only after the asexual parasitemia has peaked.

Host receptors and parasite ligands involved in the adhesion and sequestration of gametocytes in the vascular endothelium offer potential targets for potential transmission-blocking vaccines [115]. Specific host receptors involved in the general sequestration of infected erythrocytes include: CD36 (a glycoprotein), vascular cell adhesion molecule-1 (VCAM-1), intracellular adhesion molecule-1 (ICAM-1) and thrombospondin (TSP) [116]. All but VCAM-1 have been found to bind to the parasite ligand PfEMP-1 [117].

These molecules might also be targeted by immunotherapies or moieties that specifically bind or otherwise inhibit them. Thus, agents that interfere with the surface antigens expressed on young, sequestering gametocytes could impede normal gametocyte development in *P. falciparum*.

Some parasite ligands, particularly those associated with the sequestration of Stage II and IV gametocytes in bone marrow, have yet to be characterized although putative host receptors include CD49c, CD164, CD166 and ICAM-1 [118]. It may be that ligand candidates or their associated markers could be identified indirectly through genomic analysis [115]. Some of the more promising targets include:

1) pfEMP1 (*Plasmodium falciparum* erythrocyte membrane protein-1) – long known to be an important parasite ligand [119];
2) STEVOR (subtelomeric variable open reading frame) protein family – found on the surface of mid to late stage (III-V) gametocytes [115];
3) CPW-WPC proteins (including PF11.0045) – may function as adhesins. Transcription peaks in gametocytes [115];
4) PF13_0006 – a gene transcript of the RIFIN (repetitive interspersed) protein family that is abundant in mature (Stage V) gametocytes [120].

Interference with or reversal of adhesion might be accomplished with conjugates incorporating monoclonal antibodies or segments of peptide ligands, similar to approaches suggested for prospective treatments of severe malaria involving blockage of cerebral capillaries [121,122].

One clinical study [121] attempting to use 400 mg/kg high-titer antibodies to reverse cytoadherence of erythrocytes infected with asexual stages failed to affect adherence, possibly because only one-fifth of the concentration of antibodies necessary to reverse adherence *in vitro* was administered to patients. It has been suggested that excessively high levels of antibody (2X the normal presence in peripheral blood) may be necessary to successfully reverse sequestration in the systems thus far evaluated [122]. It is not clear how well the sexual stages of *Plasmodium* would respond to such an approach. Immunoliposomes have also been suggested as a possible mode of drug delivery for monoclonal antibody conjugates targeting adhesion molecules [123].

Molecules involved in adhesion may also play other roles in gametocyte development and sporogony that offer alternative mechanisms of interference. For example, the expression of lectin adhesive-like proteins (PbLAP1, 2, 4 and 6) have been shown to be critical for oocyst maturation and sporozoite formation in *P. berghei* [124,125]. This expression occurs from female nucleus-derived mRNA prior to the expression of proteins from male-derived sequences, likely during the transition from gametocyte to ookinete [125]. *P. falciparum*-specific analogues (PfLAP 1 and 4) of these molecules have also been shown to be necessary for successful sporozoite infection of mosquito salivary glands [126].

An array of LAP-related PfCCP adhesion proteins (PfCCP1, PfCCP2, PfCCP3) occur co-dependently in the parasitophorous vacuole of the gametocyte plasma membrane of *P. falciparum* [127]. They are expressed in Stages II-V of gametocytogenesis as well as during the early part of gametogenesis. Disruption of PfCCP2 and PfCCP3 genes interrupts sporogony by interfering with the transition of sporozoites from oocysts to mosquito salivary glands [126]. PfCCP4 has been found to co-localize and form a complex with the gametocyte specific surface antigen Pfs230, but does not appear to be essential for sexual stage development [127].

## 8. Unintended Consequences: Drugs that Extend Gametocyte Carriage

Some drugs have been known to stimulate gametocyte development and/or prolong the infectiousness of treated individuals. Such treatments have the potential to counteract burden reductions at the community level by increasing the rate of new infections.

Treatment with chloroquine [128] or sulfadoxine-pyrimethamine (SP) [129,130], for example, have long been known to increase the number of gametocytes circulating in the peripheral blood, possibly due to the release of sequestered parasites or through natural periodicity of parasite development.

Treatment of parasitemic but asymptomatic individuals, however, was found not to increase gametocyte carriage [131]. Thus, the use of SP for Intermittent Preventive Therapy (IPT) in infants and pregnant women should not affect malaria transmission, assuming the absence of clinical infection at the time of treatment.

This apparent escape of parasite genotypes surviving selection with SP would appear to accelerate the evolution of drug resistance [132], particularly for prophylactic regimens focused on infants (IPTi) and in areas where resistance has not emerged, as suggested by certain mathematical models [133], however some recent findings have appeared to allay some of these concerns. In Mali, one full year of intensive IPT treatment using SP resulted in no detectable increase in molecular markers of SP resistance [134]. Furthermore, SP used as IPT seems to impair the infectivity of gametocytes to mosquitoes [135,136], perhaps by interfering with the maturation process, resulting in malformed gametocytes that fail to exflagellate.

Ideally, a replacement therapy for IPT would employ a drug or drugs that did not increase gametocyte carriage and prolong the infectivity of treated individuals, under any circumstances. Certain prospective front-line treatments, such as dihydroartemisinin-piperaquine, while reducing overall gametocyte carriage appear less active against gametocytes than standard ACTs such as AS-AQ [137]. These properties also raise concerns, as the ideal therapy as we strive towards elimination should impose greater restraints on transmission, not less.

## 9. Conclusions

No ideal transmission-blocking antimalarial drug has yet been developed, though several of the prospects discussed show great promise. The ideal drug should be active against both sexual and asexual stages, should affect mature gametocytes as well as sequestered developing forms. It should also exhibit persistent and stable pharmacokinetics and pharmacodynamics to compensate for delays in parasite development, release from sequestration or response to therapies. This follows, of course, after the array of other necessary and desirable properties that any drug should have, including safety, bioavailability, and lack of existing or easily selected for resistance.

Under certain constraints, drugs active against sexual stages might help slow the development of resistance if included as part of a combination therapy, though both drugs would need to achieve a certain minimal clinical efficacy against asexual parasites relative to the treatment rate in the human population [138]. Modeling suggests that gametocytocidal monotherapies, gametocytocides with low activity against asexual stages and high coverage rates are more likely to accelerate resistance through the forces of natural selection [139].

Modes of action against asexual parasites that differ between partner drugs would assist the cause of resistance avoidance by allowing one drug to kill parasites that do not respond therapeutically to its partner. Transmission blocking effects in at least one element of a combination drug could then slow resistance by inhibiting the transmission and subsequent propagation of resistant forms. This approach would be most likely to be successful in low transmission areas where stochastic effects are more likely to come into play.

Other models suggest that in certain settings, even therapies with modest transmission blocking activity, such as current front-line ACTs, might be capable of achieving transmission reductions equivalent to that achieved by insecticide-treated nets [139]. Simulations predict that proportional reductions would be greatest (53%) where parasitemias were sparsest (3.7% slide positivity) and least (11%) where transmission was highest (57.1% slide positivity). In areas of high transmission, more of this reduction was due to the prophylactic effect of long-acting therapies, rather than their gametocytocidal properties, due to the higher frequency of re-infection.

In low transmission settings, where the force of infection is modest and the risk or re-infection is low, it can be safely assumed, however, that an ACT bearing a component molecule with more complete activity against the sexual stages of parasites would show an even greater impact on transmission at the community level than currently available ACTs.

Thus, transmission-blocking drugs seem best suited for fringe areas where malaria transmission is light or in locations that are entering the end-stages of elimination, and where mass treatment seems most feasible. By reducing the amount of inoculum available to infect vector mosquitoes, gametocytocidal drugs given as part of a mass presumptive treatment would seem a powerful complement to the integrated efforts that would be necessary to achieve the local elimination of falciparum malaria.

## Figures and Tables

**Table 1:** Developmental stages of gametocytes [14,15].

**Stage**	**Days**	**Shape**	**Cell Cycle Phase**
I -	0-2	Boat	G1
II -	1-4	D	S
III -	2-8	D	G2A
IV -	6-10	Spindle	G2B
V -	9-23	Crescent	G2B
